# Associations of municipality-level income and racial segregation with individual-level tuberculosis treatment outcomes in Brazil: a nationwide cohort study (2010–2019)

**DOI:** 10.1136/jech-2024-223465

**Published:** 2025-07-07

**Authors:** Qanisha Hall, José Firmino de Sousa Filho, Joanna MN Guimarães, Deborah C Malta, Natalia Cristina Romero-Sandoval, Sally Hargreaves, Ligia Kerr, Gervasio F Santos, Elizabeth B Brickley, Enny S Paixão, Maurício L Barreto, Julia M Pescarini, on behalf of the Unit on the Social and Environmental Determinants of Health Inequalities (SEDHI)

**Affiliations:** 1 Faculty of Epidemiology and Population Health, London School of Hygiene and Tropical Medicine, London, UK; 2 Centre for Data and Knowledge Integration for Health (CIDACS), Instituto Goncalo Moniz, Fundacao Oswald Cruz (Fiocruz), Salvador, Bahia, Brazil; 3 Escola de Enfermagem, Universidade Federal de Minas Gerais, Belo Horizonte, Minas Gerais, Brazil; 4 Faculdade de Ciencias Medicas de la Salud y Vida, Universidad Internacional del Ecuador, Quito, Ecuador; 5 Institute for Infection and Immunity, School of Health & Medical Sciences, City St George’s, University of London, London, UK; 6 Centro de Ciencias da Saude Comunitaria, Universidade Federal do Ceará, Fortaleza, Ceará, Brazil; 7 Faculdade de Economia, Universidade federal da Bahia (UFBA), Salvador, Bahia, Brazil; 8 The Unit on the Social and Environmental Determinants of Health Inequalities (SEDHI), Glasgow, UK

**Keywords:** TUBERCULOSIS, Health inequalities, HOUSING, POVERTY, TREATMENT OUTCOME

## Abstract

**Background:**

Residential segregation is considered a social determinant of health, but there is limited evidence of its impact on tuberculosis (TB). We investigated the associations between municipality-level income and racial segregation and TB treatment outcomes in Brazil.

**Methods:**

We studied nationwide registries of new TB cases between 1 January 2010 and 31 December 2019. TB treatment was dichotomised as unfavourable (ie, loss to follow-up, modification of treatment regimen, treatment failure and death) and favourable (ie, cured/treatment completion). We assessed individuals' municipality-level income and racial segregation (ie, dispersion of household heads earning ≤half versus those earning >half minimum wage; and of household heads identifying as black or brown/mixed race (*Pardo/a*) versus white). Logistic regression adjusted for sociodemographic and clinical variables was used to estimate the OR of experiencing an unfavourable treatment outcome associated with segregation overall and by self-identified race/ethnicity.

**Results:**

Individuals living in highly economically and racially segregated municipalities (fifth versus first quintiles) were more likely to have an unfavourable TB treatment outcome (income segregation: adjusted OR 1.34 (95% CI 1.31 to 1.37); racial segregation: 1.13 (0.94 to 1.36)). Living in municipalities of higher income segregation (third, fourth and fifth quintiles) was associated with higher unfavourable TB treatment outcomes in all self-identified racial groups (fifth quintile: white 1.25 (0.96 to 1.64); black 1.42 (1.15 to 1.74); brown/mixed 1.37 (1.20 to 1.56); Asian=1.30 (1.00 to 1.69) and Indigenous 1.37 (1.00 to 1.87)).

**Conclusions:**

Living in highly income and racially segregated environments is associated with unfavourable TB treatment outcomes for all self-identified races in Brazil. TB programmes should account for segregation as a barrier to TB treatment completion.

**WHAT IS ALREADY KNOWN ON THIS TOPIC**

- There has been previous research showing that residential segregation is associated with poorer health status in high-income (eg, the USA) and in middle-income countries (eg, Brazil), and evidence of segregation is associated with higher incidences of tuberculosis (TB) in the US context.

**WHAT THIS STUDY ADDS**

- This study has found new evidence that living in municipalities with higher levels of income and racial segregation is associated with a higher risk of unfavourable TB treatment outcomes in Brazil and that this is likely to be consistent among individuals of different races/ethnicities.

**HOW THIS STUDY MIGHT AFFECT RESEARCH, PRACTICE OR POLICY**

- This study provides preliminary steps into research on residential segregation and TB health outcomes in Brazil. Residential segregation should be considered as a barrier to successful TB treatment outcomes, with TB policies taking this into consideration.

## Introduction

 Tuberculosis (TB) is a treatable and curable disease, yet in 2023, it once again became the leading cause of death from a single infectious agent globally.^1^ More than 95% of TB deaths occur in low- and middle-income countries, such as Brazil, where individuals face higher risks of infection as well as socioeconomic and structural barriers to access treatment.^2^ In 2021, Brazil^1^ reported an overall TB incidence rate of 48 cases per 100 000 people,^1^ and a cure rate of 70%. The 12% treatment dropout rate and 8% fatality rate are below the acceptable rates set by the WHO’s 2022 target of ≥85% cure rates and <5% treatment dropout.^3 4^

TB incidence and treatment outcomes are linked to inequalities and poor living conditions.^5^ Several studies have shown that socioeconomic position, educational attainment, race and other social markers are associated with unfavourable TB outcomes.^6^,^8^ It has been suggested that this is due to increased barriers in accessing health services, affordable transportation to a health unit and/or time off work to meet appointments.^4^ Therefore, it is imperative to explore the factors hampering TB treatment to reduce the burden of the disease.

A hypothesised risk factor for TB adverse treatment outcomes has been residential segregation, which is the uneven distribution of people within a specified geographical area based on sociodemographic/cultural factors such as their income levels or race.^9^ Income segregation has resulted in affluent and low-income individuals living in distinct areas with limited overlap, which has implications for social mobility, resource access and overall quality of life.^10^ Furthermore, a visible manifestation of structural racism is racial segregation, which reinforces health inequalities through poor housing and economic opportunities.^11^

In Brazil, there have not been any explicit policies that have created spatial segregation based on race, but historically, disparities in the concentration of economic and political power have led to a disproportionate number of black Brazilians residing in areas with less economic development.^12 13^ The income and spatial segregation in Brazil are also reflected in the inequality of educational, healthcare and transportation services, which are poorer in favelas and more segregated municipalities.^14^

To better understand the relationship between income and racial residential segregation and unfavourable TB treatment outcomes, we used nationwide, administratively collected TB registry data from Brazil from 2010 to 2019.

## Methods

### Study design and study setting

We conducted a cohort study with all individuals newly diagnosed with TB in Brazil between 1 January 2010 and 31 December 2019. We used de-identified individual data derived from Brazil’s nationwide Information System for Notifiable Diseases (*Sistema de Informação de Agravos de Notificação*, SINAN) on TB.^15^

Brazil is a large country in Latin America, with an area of 8 510 417.77 km^2^ and a population of 203 080 756 based on the 2022 population census.^16^ The population density is 23.86 inhabitants/km^2^ and Brazil has 5570 municipalities, with 87.4% of people living in urban areas.^16^ The largest municipality is Altamira, with an area of 159 533.31 km^2^ and a population density of 0.79 inhabitants/km,^2^ and the smallest municipality is Santa Cruz de Minas, with 3.56 km^2^ and 2274.61 inhabitants/km^2^.^16^

### Data sources

We extracted data on clinical diagnosis and treatment follow-up from SINAN-TB registries on 11 May 2023.^17^ All extracted information was recorded on the TB notification form by health professionals during a clinical visit for suspected TB and included (1) sociodemographic factors such as self-identified race/ethnicity, age, region of residence, whether the individual is part of a TB prioritised group (ie, international migrants, persons deprived of liberty, health professionals, persons experiencing homelessness) and/or household receipt of government cash transfer benefits (ie, *Bolsa Família*); (2) behavioural information on tobacco, alcohol and drug use; (3) comorbidities including diabetes, HIV coinfection, mental health/cognitive developmental conditions or other health conditions; (4) diagnostic and treatment information on the TB case including results from smear microscopy, X-rays, HIV testing, sputum culture, sensitivity tests towards medications, histopathology, molecular rapid TB tests and participation in directly observed therapy short course (DOTS) and (5) TB treatment outcome.

We also extracted information on the Residential Segregation Index, composite measures of income and racial segregation in Brazil established using the dissimilarity index based on the most recent available census data, the 2010 Brazilian Census.^18^ Dissimilarity indices were calculated to measure the dispersion of household heads earning ≤half minimum wage versus earning >half minimum wage within a municipality, while racial segregation was measured by the dispersion of black and brown/mixed (*Pardo/a*) household heads from white household heads within a municipality. The Residential Segregation Index is a continuous index with values on a 0 to 1 scale, with the maximum value of 1 representing more household segregation based on the race and income characteristics of household heads.

### Variables

Individuals with TB were linked to racial and income segregation data based on their municipality of residence. The variables for income and racial segregation were categorised into quintiles (online supplemental table 3, online supplemental figure 1A, online supplemental figure 1B). The first quintile represents evenness between the social groups and little to no segregation, and the fifth quintile represents the highest segregation between the groups. The secondary exposure in the analysis was self-identified race/ethnicity for individuals with TB in five categories (white, black, brown/mixed race, Asian and Indigenous).

Our main outcome was TB treatment outcome, which was dichotomised into favourable outcomes (ie, cured/treatment completion) or unfavourable treatment outcomes. Unfavourable treatment outcomes included loss to follow-up from healthcare unit, change in TB treatment regimen, treatment failure (defined by SINAN as positive sputum smear or culture at 4 months or for 2 consecutive months after the fourth month of TB treatment initiation), mortality during TB treatment from any other cause and mortality from TB.

### Participants

Newly diagnosed cases of TB were individuals with TB notified as a new case between 2010 and 2019, excluding relapses. We excluded individuals (1) initially registered with TB but later diagnosed with other non-TB diseases, (2) without known treatment outcomes, (3) those who transferred to another healthcare facility prior to treatment completion, (4) with drug-resistant TB (TB-DR) recorded as the outcome as there was no indication if TB-DR was primary or acquired during the course of treatment. These individuals were excluded due to limited data on covariates, which would increase the amount of missingness in the study.

### Analysis

Logistic regression models with clustered-robust SEs accounting for the municipality of residence were conducted to explore the relationship between each exposure of residential segregation and unfavourable TB treatment outcomes. First, we attained the association of residential segregation and treatment outcomes using a minimally adjusted model with a priori factors, age and sex, to provide an estimate that excludes adjustment for potential intermediates (online supplemental figure 2). Following this, multivariate logistic regression models were fitted for each exposure and fully adjusted for age, sex, education, experiencing homelessness, HIV, alcohol abuse, illicit drug use, tobacco use, clinical form of TB, diabetes, mental health/cognitive developmental conditions and DOTS. The conceptual framework by Maciel and Reis-Santos (2015) and current literature on TB health outcomes were used to identify the potential confounding variables above, as logistic regression analyses show that these covariates were associated with TB treatment outcomes.^7 19^ The missing indicator method was used for covariates with incomplete data. For each one of these, we identified whether there was an association between the covariates with missing data and either the exposures or outcome at a significance level of 0.05, and therefore, missing not at random variables were included in the analysis (online supplemental table 1). We also explored an association between residential segregation and loss to follow-up as a TB treatment outcome separately (online supplemental table 4). Lastly, we explored how racial/ethnic self-identification of individuals with TB influences the relationship between residential segregation and treatment outcomes by including interaction terms between both. Individuals racially identifying as Asian or Indigenous were excluded from this analysis on interaction with racial segregation, as the municipality base index only measures racial segregation of black, brown/mixed race and white populations in Brazil. We used the likelihood ratio test to test for a linear trend between TB health outcomes and income and racial segregation quintiles. Likelihood ratio tests were used to analyse the interaction between municipality-level household segregation and race/ethnicity, and the stratum-specific ORs were calculated. Analyses were conducted with the statistical software STATA/SE V.17.0 (College Station, TX, USA).

## Results

**Figure 1:**
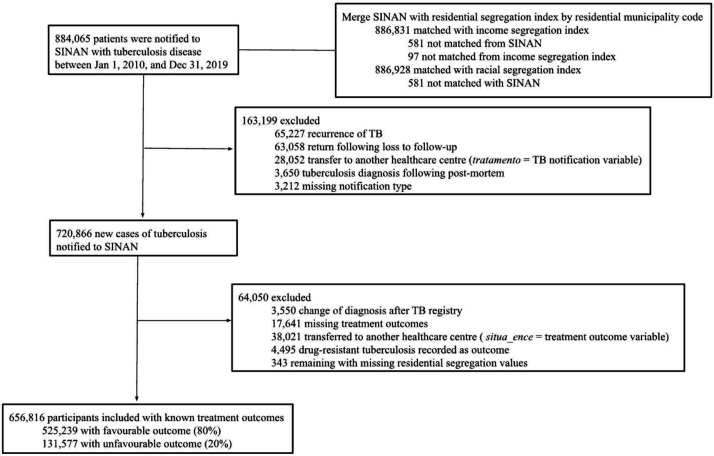
Participant selection in our cohort. SINAN, Sistema de Informação de Agravos de Notificação; TB, tuberculosis.

We studied 656 816 newly diagnosed TB cases, of whom 20.0% (131 577/656 816) had unfavourable treatment outcomes (figure 1). TB cases mostly included individuals who were 18–39 years (n=315 172, 48%), male (n=442 363, 67.4%), identified as brown/mixed race (n=2 97 206, 45.3%), held a high school level of education (n=180 348, 32.5%) and lived in the Southeast region of Brazil (n=307 901, 46.9%) (table 1).

**Table 1:** Baseline distribution of study sample and treatment outcomes for TB patients in Brazil between 1 January 2010 to 31 December 2019 (n=6 56 816)

Covariates (n=656 816)	Favourable outcome n=525 239 (%)	Unfavourable outcome n=131 577 (%)	SD*
Socioeconomic and demographic			
Age (years)			−0.16
≤17	35 491 (6.8%)	4705 (3.6%)	
18–39	253 541 (48.3%)	61 631 (46.8%)	
40–64	190 700 (36.3%)	48 397 (36.8%)	
≥65	45 462 (8.7%)	16 832 (12.8%)	
Missing	45 (0.01%)	12 (0.01%)	
Sex			−0.17
Female	179 552 (34.2%)	34 857 (26.5%)	
Male	345 652 (65.8%)	96 711 (73.5%)	
Missing	35 (0.01%)	9 (0.01%)	
Race			−0.06
White	177 800 (36.5%)	39 300 (32.6%)	
Black	62 287 (12.8%)	18 913 (15.7%)	
Asian	4119 (0.8%)	915 (0.8%)	
Brown/mixed	236 993 (48.6%)	60 213 (50%)	
Indigenous	6250 (1.2%)	1054 (0.8%)	
Missing	37 790 (7.19%)	11 182 (8.50%)	
Education			0.001
No education	21 439 (4.1%)	6748 (5.1%)	
Primary school or less (<5 years of education)	88 468 (16.8%)	23 859 (18.1%)	
Junior high school (5–9 years of education)	122 929 (23.4%)	32 939 (25%)	
High school (≥10 years of education)	155 970 (29.7%)	24 378 (18.5%)	
Missing	136 433 (25.9%)	43 653 (33.2%)	
Region (residence)			−0.05
North	59 296 (11.3%)	13 882 (10.6%)	
Northeast	132 358 (25.2%)	32 387 (24.6%)	
Southeast	247 911 (47.2%)	59 990 (45.6%)	
South	61 661 (11.7%)	19 020 (14.5%)	
Central west	24 013 (4.6%)	6298 (4.8%)	
Missing	0 (0%)	0 (0%)	
Deprivation of liberty			−0.06
Yes	35 437 (6.8%)	5500 (4.2%)	
No	281 387 (53.6%)	72 775 (55.3%)	
Missing	208 415 (39.7%)	53 302 (40.5%)	
Experiencing homelessness			0.05
Yes	4503 (0.9%)	5384 (4.1%)	
No	310 584 (59.1%)	72 539 (55.1%)	
Missing	210 152 (40%)	53 654 (40.8%)	
Government cash transfers			−0.04
Yes	15 191 (2.9%)	3440 (2.6%)	
No	147 544 (28.1%)	38 475 (29.2%)	
Missing	362 504 (69%)	89 662 (68.1%)	
Comorbidities and clinical characteristics			
HIV			
Positive	35 386 (6.7%)	25 444 (19.3%)	
Negative	375 483 (71.5%)	65 171 (49.5%)	0.05
Missing	114 370 (21.8%)	40 962 (31.1%)	
Clinical form of TB			
Pulmonary	438 313 (83.5%)	108 329 (82.3%)	
Extrapulmonary	73 386 (14%)	16 685 (12.7%)	−0.07
Pulmonary+extrapulmonary	13 534 (2.6%)	6523 (5%)	
Missing	6 (0%)	40 (0.03%)	
Alcohol abuse			0.15
Yes	68 426 (13%)	30 484 (23.2%)	
No	420 643 (80.1%)	88 551 (67.3%)	
Missing	36 170 (6.9%)	12 542 (9.5%)	
Tobacco use			0.04
Yes	56 709 (10.8%)	20 041 (15.2%)	
No	258 403 (49.2%)	56 420 (42.9%)	
Missing	210 127 (40%)	55 116 (41.9%)	
Illicit drug use			0.07
Yes	32 410 (6.2%)	16 640 (12.7%)	
No	281 542 (53.6%)	59 491 (45.2%)	
Missing	211 287 (40.2%)	55 446 (42.1%)	
Mental health/cognitive developmental conditions			−0.07
Yes	10 377 (1.9%)	3717 (2.8%)	
No	476 282 (90.7%)	114 205 (86.8%)	
Missing	38 580 (7.4%)	13 655 (10.4%)	
Diabetes			−0.09
Yes	38 824 (7.4%)	8769 (6.7%)	
No	448 061 (85.3%)	109 504 (83.2%)	
Missing	38 354 (7.3%)	13 304 (10.1%)	
Initiating DOTS			−0.17
Yes	198 565 (37.8%)	38 188 (29.0%)	
No	110 766 (21.1%)	31 348 (23.8%)	
Missing	215 908 (41.1%)	62 041 (47.2%)	

* SD is the standardised difference of the covariates distribution between individuals with favourable and unfavourable outcomes.

DOTS, directly observed therapy short course; SMD, standardised mean difference; TB, tuberculosis.

**Figure 2:**
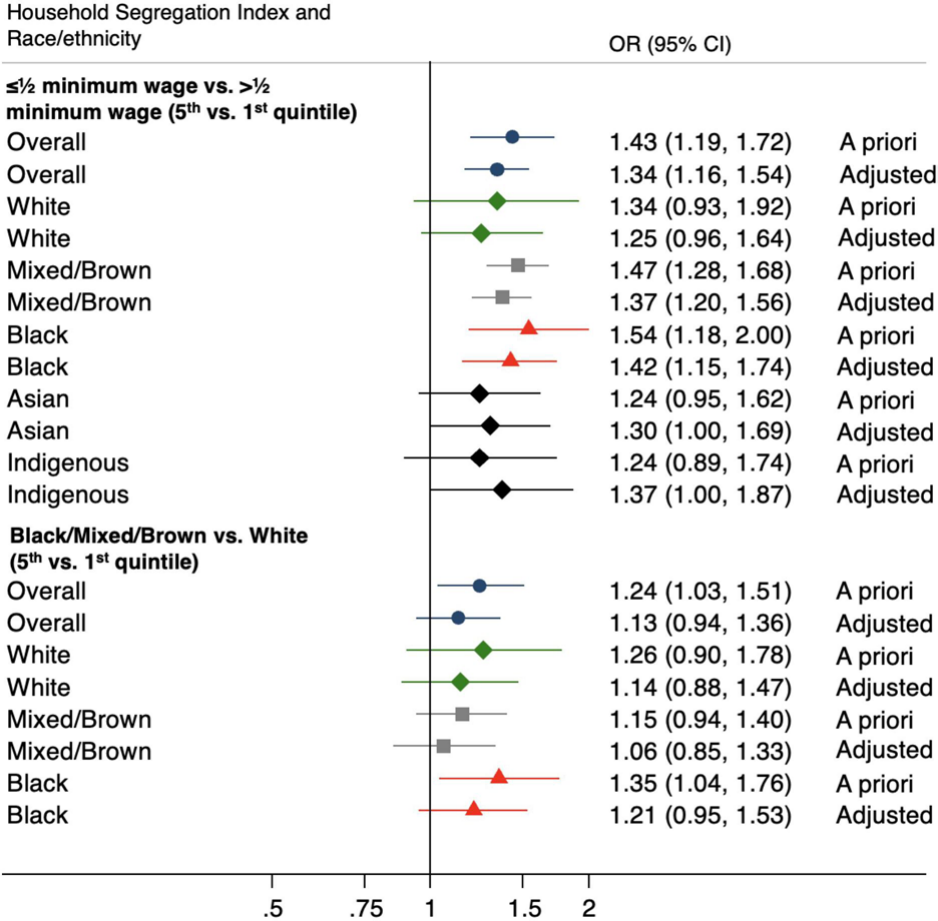
Association between household segregation and unfavourable TB treatment outcome stratified by individual’s race/ethnicity (fifth versus first quintile) (n=656 816). A priori analysis included age and sex. Adjusted analysis included age, sex, education, experiencing homelessness, HIV, alcohol abuse, illicit drug use, tobacco use, clinical form of TB, diabetes, mental/cognitive developmental conditions and DOTS. DOTS, directly observed therapy short course; TB, tuberculosis.

The minimally adjusted analysis for a priori variables showed evidence that living in municipalities with higher income segregation (ie, third, fourth and fifth quintiles of higher segregation) and higher racial segregation (fourth and fifth quintiles) lead to higher odds of an unfavourable TB treatment outcome (table 2, figure 2). After adjusting for sociodemographic and clinical covariates, we found evidence that living in municipalities with higher income segregation was associated with a higher likelihood of experiencing an unfavourable TB outcome compared with those living in municipalities with low segregation (third quintile: OR 1.14 (95% CI 1.03 to 1.25); fourth quintile: 1.27 (1.08 to 1.50); fifth quintile: 1.34 (1.16 to 1.54)) (table 2). Similarly, we found an association between racial segregation and unfavourable TB outcomes, which were mainly concentrated in those living in highly segregated municipalities (fourth quintile: OR 1.18 (95% CI 1.02 to 1.37); fifth quintile: 1.13 (0.94 to 1.36)).

**Table 2:** Crude and multivariable logistic regression between household income and racial segregation, and TB treatment outcome (n=656 816)

Residential segregation indices		OR (95% CI)*	Adjusted OR (95% CI)†
Income segregation (N, % unfavourable TB treatment outcomes)			
≤half minimum wage versus >half minimum wage	First quintile (23,608, 17.94%)	1.00 (base)	1.00 (base)
	Second quintile (23,637, 17.96%)	0.99 (0.90 to 1.09)	0.99 (0.91 to 1.08)
	Third quintile (26,534, 20.17%)	1.14 (1.01 to 1.29)	1.14 (1.03 to 1.25)
	Fourth quintile (29,305, 22.27%)	1.23 (1.09 to 1.38)	1.27 (1.08 to 1.50)
	Fifth quintile (28,493, 21.66%)	1.43 (1.19 to 1.72)	1.34 (1.16 to 1.54)
Racial segregation			
Black and mixed/brown versus white	First quintile (24,753, 18.81%)	1.00 (base)	1.00 (base)
	Second quintile (24,699, 18.77%)	1.00 (0.91 to 1.10)	1.01 (0.93 to 1.11)
	Third quintile (25,256, 19.19%)	1.02 (0.89 to 1.17)	0.97 (0.87 to 1.08)
	Fourth quintile (28,207, 21.44%)	1.17 (1.03 to 1.33)	1.18 (1.02 to 1.37)
	Fifth quintile (28,662, 21.78%)	1.24 (1.03 to 1.51)	1.13 (0.94 to 1.36)

* Including a priori (age, sex).

† Adjusted for age, sex, education, experiencing homelessness, HIV, alcohol abuse, illicit drug use, tobacco use, clinical form of TB, diabetes, mental health/cognitive developmental conditions and DOTS.

DOTS, directly observed therapy short course; TB, tuberculosis.

In the analyses stratified by race, we found higher levels of segregation to have an impact on TB treatment outcome in all self-identified racial groups (figure 2, online supplemental table 2). Associations by race/ethnicity were more evident and consistent in the more segregated quintiles of income. Those living in municipalities with the highest quintiles of income segregation had overall higher odds of an unfavourable outcome compared with individuals living in non-segregated municipalities (fifth quintile for white: OR 1.25 (95% CI 0.96 to 1.64); fifth quintile for black: 1.42 (1.15 to 1.74); fifth quintile for brown: 1.37 (1.20 to 1.56); fifth quintile for Asian: 1.30 (1.00 to 1.69); fifth quintile for Indigenous: 1.37 (1.00 to 1.87)).

An association was also found between high levels of income segregation (fourth quintile: 1.45 (1.42 to 1.49); fifth quintile: 1.57 (1.53 to 1.61)) and high racial segregation (fourth quintile: 1.25 (1.22 to 1.28); fifth quintile: 1.22 (1.19 to 1.25)) with loss to follow-up treatment outcome (online supplemental table 4).

## Discussion

In our study, we found that individuals with TB who live in areas with higher levels of income or racial segregation are more likely to experience unfavourable TB outcomes compared with those living in areas with lower levels of segregation. Stratified analysis by race indicated that living in the highest quintiles of income segregation increases the odds of unfavourable TB treatment for all racial groups, which was 42% higher among black individuals and 37% higher among brown individuals. These findings align with the broader literature suggesting that areas with a larger amount of racial or income segregation have increased disparities in health outcomes compared with areas with less segregation.^20^

Residential segregation is an established determinant of adverse health outcomes in the USA context, such as TB^21^,^23^ and chronic obstructive pulmonary disease,^24^ COVID-19 mortality^25^ and gonorrhoea.^26^ When looking at TB, most research has focused on racial segregation and its association with TB incidence and transmission.^21^ Studies have also found that in areas with higher racial inequality, disparities in TB incidence and mortality are more pronounced, with increased TB risk observed among immigrants, black and/or Hispanic individuals.^21 23^ Furthermore, a cross-sectional study on TB incidence in the state of Michigan found evidence of black–white racial inequalities in TB incidence rates, with higher rates among black individuals in Detroit.^23^

Our findings also contribute to the emerging evidence that segregation can also lead to poorer health outcomes in Brazil.^27^,^29^ Segregation has been found to be linked to poorer self-rated health across Brazilian cities—higher levels of income segregation were associated with poorer health across all racial groups, with stronger associations for black and brown individuals.^27^ In addition, individuals living in municipalities with higher rates of income segregation were associated with up to 18% higher breast cancer mortality,^28^ and up to 17% higher COVID-19 mortality.^29^ It is important to note that poverty can also be used as a partial indicator of residential segregation in Brazil, and poorer socioeconomic variables such as higher rates of unemployment and household crowding in municipalities have been found to be associated with higher TB incidence rates.^30^ However, no studies on residential segregation and TB were previously available from Brazil or Latin American countries due to the absence of a residential segregation measure. Our findings, therefore, add to the body of evidence suggesting that both income and racial residential segregation may contribute to poorer TB treatment outcomes, especially among historically racialised groups.

We hypothesise that segregation may contribute to unfavourable TB treatment outcomes due to barriers in accessing health services and variations in the quality of care based on residential location. Segregation perpetuates geographical manifestations of inequality between different social groups in Brazil, reinforcing sociospatial differences in access to health-based resources.^28^ Previous qualitative studies have noted barriers to TB treatment in various countries, such as Indonesia and Ghana, where it has been reported that the main barriers to treatment completion were a lack of knowledge of the free-of-charge national TB programme, inaccessibility to qualified TB care and long distance to health services.^31^,^33^

In most Brazilian cities, it was found that low-income and black individuals experience poor access to healthcare facilities due to geographical distance and dependence on public transportation services.^34^ Additionally, Coube *et al* (2023) found that unmet healthcare needs in Brazil (2013–2019) were concentrated among lower-income groups, primarily due to affordability. Their analysis showed that dual coverage by wealthier individuals (public and private) versus the poor’s reliance on Brazilian Universal Healthcare System (SUS) alone has widened health inequalities.^35^ In addition, geographical segregation also perpetuates disparities in access to other social determinants of health, such as education, employment and housing infrastructure.^36^ The isolation of economically vulnerable people or racially marginalised groups can also lead to disparate funding in segregated municipalities, subsequently resulting in a reduction of effective and high-quality public health services.^18^

In light of our findings, decentralised TB treatment may reduce access barriers. Although the Brazilian Ministry of Health recommends decentralisation,^37^ studies have shown that TB care remains centralised in some units in cities like Salvador and João Pessoa. In Salvador and Recife, decentralisation to basic units was associated with easier access to TB care, whereas in João Pessoa, centralisation led to a slight increase in access difficulties due to the magnitude of centralisation in the city.^38^ In addition, housing policies like *Minha Casa*, *Minha Vida* have predominantly been used to provide adequate housing, but can also maintain or increase residential segregation and fail to improve health outcomes.^39^ In Santa Cruz do Sul (South of Brazil), the programme resulted in the movement of individuals out of their familiar environments and creating accessibility barriers to healthcare services due to longer travel distances or unfamiliarity with healthcare services.^39^

This study is the first to investigate the role of income and racial residential segregation on TB treatment outcomes in Brazil, a disease highly associated with poverty and inequality.^40^ Using nationwide administrative data provided a large sample size and power, allowing us to adjust for multiple potentially confounding factors. However, our study has some limitations. First, some of the available covariates, such as tobacco, drug and alcohol use, are self-reported and may be under-reported due to social desirability bias, although this is likely to be non-differential across race/ethnicity and municipality of residence. Second, the analysis may also be subject to unmeasured confounding, such as individual socioeconomic status (ie, income level or job type), which is both related to segregation and can impact access to TB treatment. It is also possible that some individuals with TB share several risk factors, and this could result in overadjustment. However, many factors are intrinsically linked to poverty and therefore included in the analysis. A minimally adjusted analysis, which includes adjustment for age and sex only, is included in order to show the total effect of segregation on TB outcomes. Finally, we have included a missing indicator analysis that is suggested to introduce bias. However, this is still one of the best approaches for dealing with missing data when data are not missing at random. Complete case analysis was not used in the main analysis as eight covariates were missing more than 20% of their data and because missing data were found to be associated with both the exposure and outcome and, therefore, not missing at random. So, we need to be cautious with the interpretation of the complete case analysis as this is not generalisable to most people with TB (see online supplemental table 5). Finally, it is important to note that the analyses exploring how racial segregation interacts with self-identified race have limited generalisability as the number of Asian and Indigenous people with TB in our sample was low and, therefore, it is not possible to conduct stratified analysis for these groups.

## Conclusions

Our research suggests that higher residential income and racial segregation are associated with unfavourable TB treatment outcomes in Brazil, with a particularly higher risk among individuals living in the highest segregated municipalities. This result provides a preliminary step in research on the nexus between residential segregation and TB treatment outcomes. This suggests that a structural approach is needed to improve TB care by enhancing the quality and availability of health services, expanding treatment facilities, and improving transport infrastructure and service frequency to reduce geographic and mobility-related barriers. Future research would benefit from exploring the quality of healthcare services for TB in municipalities with high income and racial segregation, as well as a more thorough adjustment for possible confounding variables. With TB remaining an important public health issue in Brazil, it is important to understand the political, social and environmental factors influencing treatment outcomes.

## Supplementary material

10.1136/jech-2024-223465 online supplemental file 1

10.1136/jech-2024-223465 online supplemental file 2

10.1136/jech-2024-223465 online supplemental file 3

10.1136/jech-2024-223465 online supplemental file 4

## Data Availability

Data are available in a public, open access repository.

## References

[1] Martins-Melo FR, Bezerra JMT, Barbosa DS (2024). The burden of tuberculosis and attributable risk factors in Brazil, 1990–2017: results from the Global Burden of Disease Study 2017. World Health Organisation. Tuberculosis.

[2] Martins-Melo FR, Bezerra JMT, Barbosa DS (2020). The burden of tuberculosis and attributable risk factors in Brazil, 1990-2017: results from the Global Burden of Disease Study 2017. Popul Health Metr.

[3] (2022). Global tuberculosis report 2022.

[4] Lucena LA de, Dantas GB da S, Carneiro TV (2023). Factors Associated with the Abandonment of Tuberculosis Treatment in Brazil: A Systematic Review. Rev Soc Bras Med Trop.

[5] Basta PC, Marques M, Oliveira RL de (2013). Desigualdades sociais e tuberculose: análise segundo raça/cor, Mato Grosso do Sul. Rev Saúde Pública.

[6] Albuquerque M de FPM de, Ximenes RA de A, Lucena-Silva N (2007). Factors associated with treatment failure, dropout, and death in a cohort of tuberculosis patients in Recife, Pernambuco State, Brazil. Cad Saúde Pública.

[7] Chenciner L, Annerstedt KS, Pescarini JM (2021). Social and health factors associated with unfavourable treatment outcome in adolescents and young adults with tuberculosis in Brazil: a national retrospective cohort study. Lancet Glob Health.

[8] Viana PV de S, Gonçalves MJF, Basta PC (2016). Ethnic and Racial Inequalities in Notified Cases of Tuberculosis in Brazil. PLoS ONE.

[9] Timberlake JM, Ignatov MD (2014). Residential segregation. Oxford Bibliographies.

[10] Ahmed MK, Scretching D, Lane SD (2023). Study designs, measures and indexes used in studying the structural racism as a social determinant of health in high income countries from 2000-2022: evidence from a scoping review. Int J Equity Health.

[11] White A, Thornton RLJ, Greene JA (2021). Remembering Past Lessons about Structural Racism - Recentering Black Theorists of Health and Society. N Engl J Med.

[12] Telles EE (1992). Residential Segregation by Skin Color in Brazil. Am Sociol Rev.

[13] Barber S, Ferreira A, Gripper AB (2022). Healthy and thriving: advancing anti-racism evidence and solutions to transform the health of Black communities. The Lancet.

[14] Carvalho C, Netto VM (2023). Segregation within segregation: Informal settlements beyond socially homogenous areas. Cities.

[15] Ministério da Saúde TUBERCULOSE - casos confirmados notificados no sistema de informação de agravos de notificação - Brasil. https://portalsinan.saude.gov.br/tuberculose.

[16] Instituto Brasileiro de Geografia e Estatística (2022). Censo demográfico.

[17] Sistema de Informação de Agravos de Notificação SINANWEB - tuberculose. https://datasus.saude.gov.br/transferencia-de-arquivos/.

[18] de Sousa Filho JF, Dos Santos GF, Andrade RFS (2022). Inequality and income segregation in Brazilian cities: a nationwide analysis. SN Soc Sci.

[19] Maciel EL, Reis-Santos B (2015). Determinants of tuberculosis in Brazil: from conceptual framework to practical application. Rev Panam Salud Publica.

[20] Telles EE (2004). Race in another America: the significance of skin color in Brazil.

[21] Acevedo-Garcia D (2000). Residential segregation and the epidemiology of infectious diseases. Soc Sci Med.

[22] Du Bois WEB (2023). The Philadelphia negro: a social study.

[23] Noppert GA, Clarke P, Hicken MT (2019). Understanding the intersection of race and place: the case of tuberculosis in Michigan. BMC Public Health.

[24] Woo H, Brigham EP, Allbright K (2021). Racial Segregation and Respiratory Outcomes among Urban Black Residents with and at Risk of Chronic Obstructive Pulmonary Disease. Am J Respir Crit Care Med.

[25] Franz B, Parker B, Milner A (2022). The Relationship between Systemic Racism, Residential Segregation, and Racial/Ethnic Disparities in COVID-19 Deaths in the United States. Ethn Dis.

[26] Pugsley RA, Chapman DA, Kennedy MG (2013). Residential segregation and gonorrhea rates in US metropolitan statistical areas, 2005-2009. Sex Transm Dis.

[27] Guimarães JMN, Yamada G, Barber S (2022). Racial Inequities in Self-Rated Health Across Brazilian Cities: Does Residential Segregation Play a Role?. Am J Epidemiol.

[28] Guimarães JMN, Pescarini JM, Sousa Filho JF de (2024). Income Segregation, Conditional Cash Transfers, and Breast Cancer Mortality Among Women in Brazil. *JAMA Netw Open*.

[29] Sousa Filho JF de, Silva UM, Lima LL (2022). Association of urban inequality and income segregation with COVID-19 mortality in Brazil. PLoS One.

[30] Pelissari DM, Rocha MS, Bartholomay P (2018). Identifying socioeconomic, epidemiological and operational scenarios for tuberculosis control in Brazil: an ecological study. BMJ Open.

[31] Appiah MA, Arthur JA, Asampong E (2024). Health service providers’ perspective on barriers and strategies to tuberculosis treatment adherence in Obuasi Municipal and Obuasi East District in the Ashanti region, Ghana: a qualitative study. *Discov Health Systems*.

[32] Aibana O, Dauria E, Kiriazova T (2020). Patients’ perspectives of tuberculosis treatment challenges and barriers to treatment adherence in Ukraine: a qualitative study. BMJ Open.

[33] Pradipta IS, Idrus LR, Probandari A (2021). Barriers and strategies to successful tuberculosis treatment in a high-burden tuberculosis setting: a qualitative study from the patient’s perspective. BMC Public Health.

[34] Tomasiello DB, Vieira JPB, Parga JPFA (2024). Racial and income inequalities in access to healthcare in Brazilian cities. J Transp Health.

[35] Coube M, Nikoloski Z, Mrejen M (2023). Inequalities in unmet need for health care services and medications in Brazil: a decomposition analysis. Lancet Reg Health Am.

[36] Leivas PHS, dos Santos AMA (2018). Horizontal inequality and ethnic diversity in Brazil: patterns, trends, and their impacts on institutions. Oxf Dev Stud.

[37] Ministério da Saúde (2018). Manual de recomendações para o controle da tuberculose no Brasil. Ms.

[38] Souza MSPL, Aquino R, Pereira SM (2015). Fatores associados ao acesso geográfico aos serviços de saúde por pessoas com tuberculose em três capitais do Nordeste brasileiro. Cad Saúde Pública.

[39] Cadoná MA, Tirelli C, Coutinho Areosa SV (2016). Políticas habitacionais, segregação residencial e desigualdade no acesso às políticas públicas: uma análise a partir do acesso a serviços públicos de saúde / Housing policies, residential segregation and inequality in access to public policies: an analysis based on access to public health services. REDES.

[40] Pinto PFPS, Santos BPS dos, Teixeira CSS (2022). Avaliação de desempenho do controle da tuberculose em municípios brasileiros. Rev saúde pública.

