# Supplementary figures and images for: Associations of municipality-level income and racial segregation with individual-level tuberculosis treatment outcomes in Brazil: a nationwide cohort study (2010–2019)
Source: J Epidemiol Community Health. 2025 Jul 7;79(10):e223465. doi: 10.1136/jech-2024-223465 (PMC12505094; doi:10.1136/jech-2024-223465)

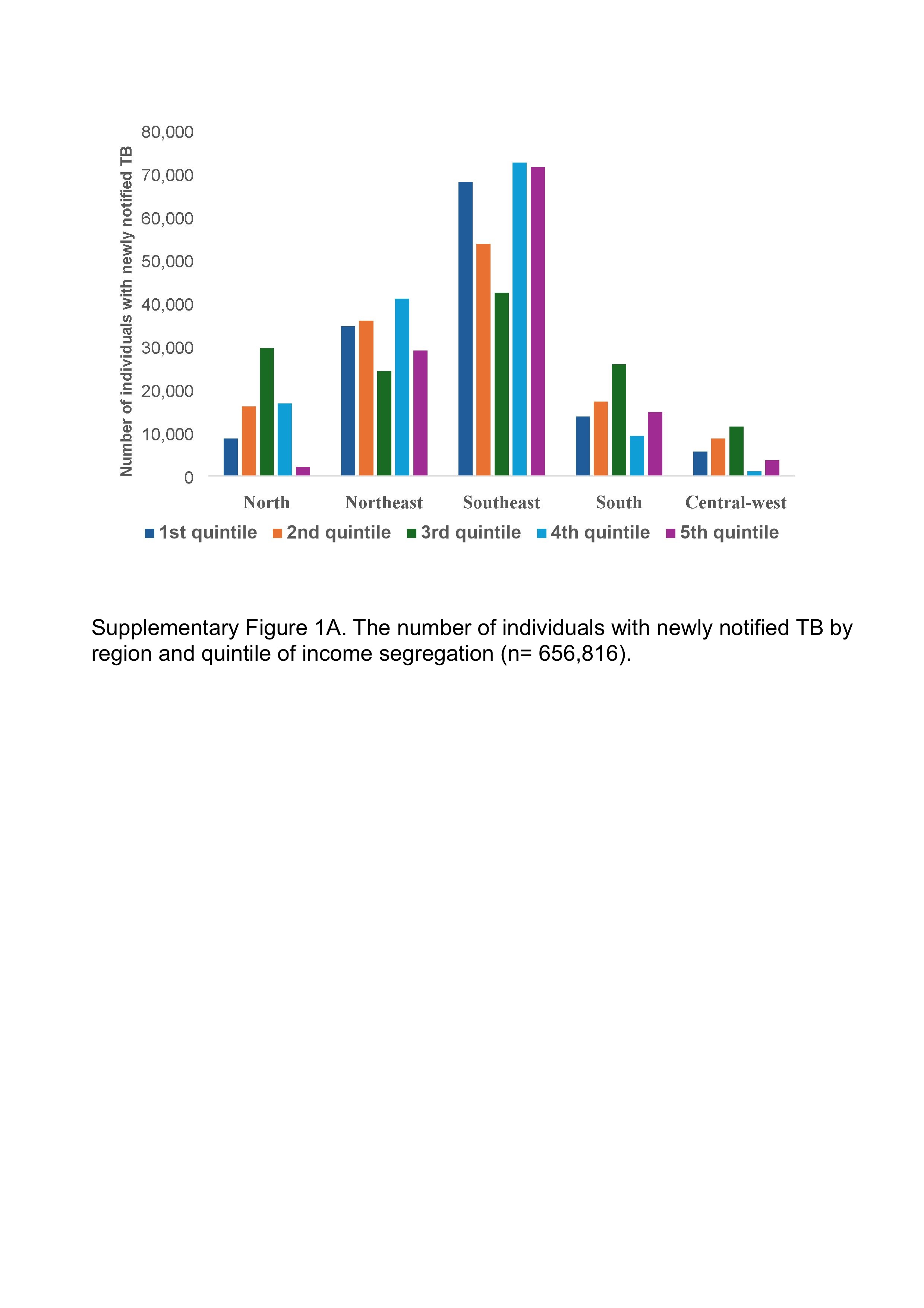

Supplement: online supplemental file 1 [file jech-79-10-s001.jpg]

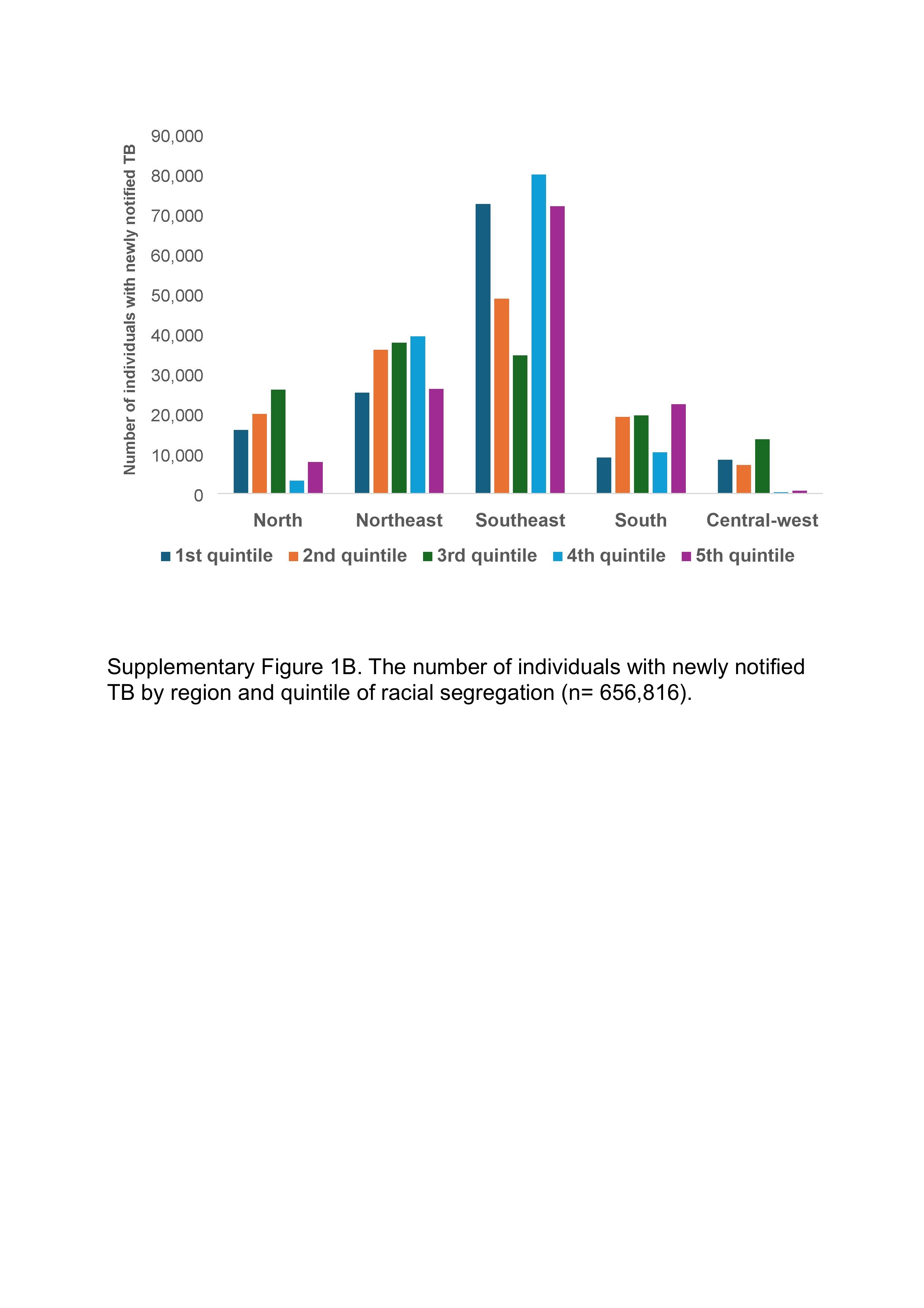

Supplement: online supplemental file 2 [file jech-79-10-s002.jpg]

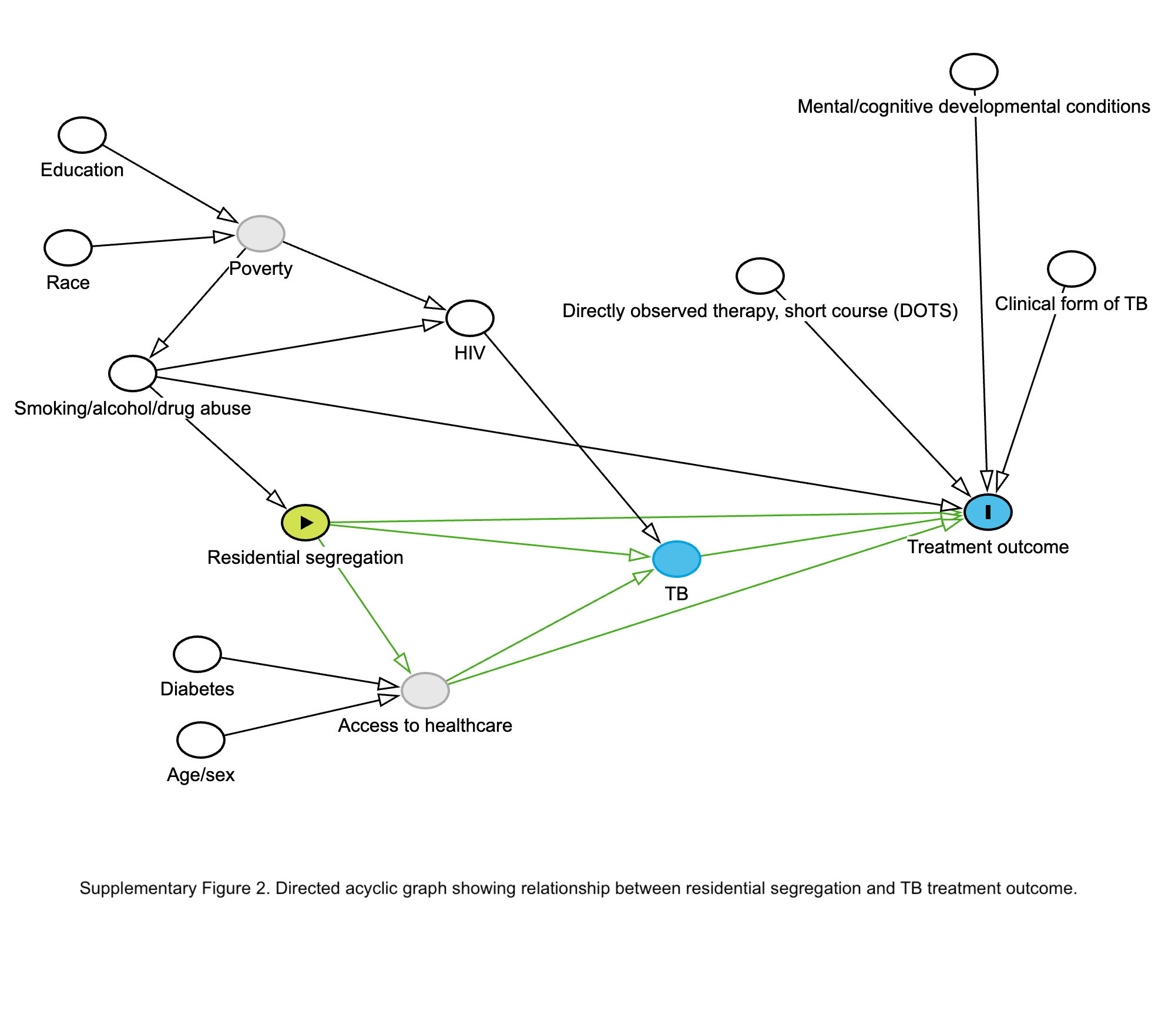

Supplement: online supplemental file 4 [file jech-79-10-s004.png]
