# Supplementary material for: Associations of municipality-level income and racial segregation with individual-level tuberculosis treatment outcomes in Brazil: a nationwide cohort study (2010–2019)
Source: J Epidemiol Community Health. 2025 Jul 7;79(10):e223465. doi: 10.1136/jech-2024-223465 (PMC12505094; doi:10.1136/jech-2024-223465)
Supplement: online supplemental file 3 [file jech-79-10-s003.docx]

**Supplementary Table 1. Missing data analysis for covariates (n= 656,816)**

| Covariates | Missing (%) | Association with treatment outcome* | Association with racial segregation* | | | Association with income segregation* | | |
| --- | --- | --- | --- | --- | --- | --- | --- | --- |
|  |  |  | Black - White | Mixed/Brown - White | Black, Mixed/Brown - White | ≤1/2 min wage - >1/2 min wage | ≤1 min wage - >1 min wage | ≤2 min wages - >2 min wages |
| Age | 57 (0.01%) | P = 0.8483 | P<0.0001 | P<0.0001 | P<0.0001 | P = 0.0001 | P<0.0001 | P = 0.0001 |
| Sex | 44 (0.01%) | P = 0.9444 | P=0.0489 | P=0.0013 | P=0.1686 | P=0.2968 | P=0.4696 | P=0.9260 |
| Race | 48,972 (7.46%) | P<0.0001 | P<0.0001 | P<0.0001 | P<0.0001 | P<0.0001 | P<0.0001 | P<0.0001 |
| Education | 180,086 (27.42%) | P<0.0001 | P<0.0001 | P<0.0001 | P<0.0001 | P<0.0001 | P<0.0001 | P<0.0001 |
| HIV | 155,332 (23.65%) | P<0.0001 | P<0.0001 | P<0.0001 | P<0.0001 | P<0.0001 | P<0.0001 | P<0.0001 |
| Diabetes | 51,658 (7.86%) | P<0.0001 | P<0.0001 | P<0.0001 | P<0.0001 | P<0.0001 | P<0.0001 | P<0.0001 |
| Mental health/cognitive developmental conditions | 52,235 (7.95%) | P<0.0001 | P<0.0001 | P<0.0001 | P<0.0001 | P<0.0001 | P<0.0001 | P<0.0001 |
| Immigrant | 291, 877 (44.44%) | P=0.0039 | P<0.0001 | P<0.0001 | P<0.0001 | P<0.0001 | P<0.0001 | P<0.0001 |
| Homeless | 263,806 (40.16%) | P<0.0001 | P<0.0001 | P<0.0001 | P<0.0001 | P<0.0001 | P<0.0001 | P<0.0001 |
| Alcohol use | 48,712 (7,42%) | P<0.0001 | P<0.0001 | P<0.0001 | P<0.0001 | P<0.0001 | P<0.0001 | P<0.0001 |
| Tobacco use | 265,243 (40.38%) | P<0.0001 | P<0.0001 | P<0.0001 | P<0.0001 | P<0.0001 | P<0.0001 | P<0.0001 |
| Illicit drug use | 266,733 (40.61%) | P<0.0001 | P<0.0001 | P<0.0001 | P<0.0001 | P<0.0001 | P<0.0001 | P<0.0001 |
| DOTS | 277,949 (42.32%) | P<0.0001 | P<0.0001 | P<0.0001 | P<0.0001 | P<0.0001 | P<0.0001 | P<0.0001 |
| *Chi-squared p-value from logistic regression, testing the association between covariates with outcome and exposure | | | | | | | | |

**Supplementary Table 2. Analysis of effect modification by race in the association between residential segregation and treatment outcome (n= 656,816)**

|  |  | Favourable (%) | Unfavourable (%) | Stratum-specific adjusted OR  [95% CI]* |
| --- | --- | --- | --- | --- |
| White | ***≤1/2 minimum wage vs >1/2 minimum wage*** | | | |
|  | 1st | 33,949 (83.61%) | 6,655 (16.39%) | 1.00 (Base) |
|  | 2nd | 36,355 (82.78%) | 7,563 (17.22%) | 1.06 [0.98 - 1.14] |
|  | 3rd | 38,880 (82.17%) | 8,435 (17.83%) | 1.10 [1.00 - 1.20] |
|  | 4th | 35,195 (81.37%) | 8,056 (18.63%) | 1.25 [1.09 - 1.42] |
|  | 5th | 33,421 (79.55%) | 8,591 (20.45%) | 1.25 [0.96 - 1.64] |
|  | ***Black & Mixed/Brown vs. White*** | | | |
|  | 1st | 31,634 (82.94%) | 6,509 (17.06%) | 1.00 (Base) |
|  | 2nd | 36,427 (82.40%) | 7,779 (17.60%) | 1.05 [0.96 - 1.14] |
|  | 3rd | 36,523 (83.25%) | 7,351 (16.75%) | 0.96 [0.87 – 1.04] |
|  | 4th | 38,157 (81.56%) | 8,627 (18.44%) | 1.11 [0.96 - 1.30] |
|  | 5th | 35,059 (79.51%) | 9,034 (20.49%) | 1.14 [0.88 - 1.47] |
| Brown | ***≤1/2 minimum wage vs >1/2 minimum wage*** | | | |
|  | 1st | 50,038 (81.88%) | 11,070 (18.12%) | 1.00 (Base) |
|  | 2nd | 50,899 (82.27%) | 10,972 (17.73%) | 0.97 [0.87 - 1.08] |
|  | 3rd | 47,506 (79.14%) | 12,525 (20.86%) | 1.18 [1.05 - 1.34] |
|  | 4th | 52,821 (78.61%) | 14,372 (21.39%) | 1.31 [1.09 - 1.57] |
|  | 5th | 35,729 (76.01%) | 11,274 (23.99%) | 1.37 [1.20 - 1.56] |
|  | ***Black & Mixed/Brown vs. White*** | | | |
|  | 1st | 50,357 (80.95%) | 11,848 (19.05%) | 1.00 (Base) |
|  | 2nd | 48,776 (80.64%) | 11,709 (19.36%) | 1.02 [0.92 - 1.1] |
|  | 3rd | 52,163 (79.89%) | 13,131 (20.11%) | 1.07 [0.91 - 1.27] |
|  | 4th | 44,792 (77.97%) | 12,656 (22.03%) | 1.22 [1.03 - 1.45] |
|  | 5th | 40,905 (79.01%) | 10,869 (20.99%) | 1.15 [0.94 - 1.40] |
| Black | ***≤1/2 minimum wage vs >1/2 minimum wage*** | | | |
|  | 1st | 13,484 (78.90%) | 3,605 (21.10%) | 1.00 (Base) |
|  | 2nd | 12,382 (79.78%) | 3,139 (20.22%) | 0.92 [0.80 - 1.05] |
|  | 3rd | 10,525 (77.24%) | 3,101 (22.76%) | 1.08 [0.94 - 1.25] |
|  | 4th | 12,407 (76.97%) | 3,712 (23.03%) | 1.18 [0.93 - 1.49] |
|  | 5th | 13,489 (71.58%) | 5,356 (28.42%) | 1.42 [1.15 - 1.74] |
|  | ***Black & Mixed/Brown vs. White*** | | | |
|  | 1st | 13,726 (77.87%) | 3,900 (22.13%) | 1.00 (Base) |
|  | 2nd | 10,695 (78.75%) | 2,886 (21.25%) | 0.94 [0.84 – 1.06] |
|  | 3rd | 10,216 (79.28%) | 2,670 (20.72%) | 0.87 [0.75 – 1.00] |
|  | 4th | 11,515 (77.02%) | 3,435 (22.98%) | 1.09 [0.89 - 1.32] |
|  | 5th | 16,135 (72.82%) | 6,022 (27.18%) | 1.21 [0.95 - 1.53] |
| Asian | ***≤1/2 minimum wage vs >1/2 minimum wage*** | | | |
|  | 1st | 747 (82.82%) | 155 (17.18%) | 1.00 (Base) |
|  | 2nd | 779 (84.13%) | 147 (15.87%) | 0.90 [0.69 - 1.17] |
|  | 3rd | 824 (82.15%) | 179 (17.85%) | 1.05 [0.79 - 1.40] |
|  | 4th | 1,006 (80.42%) | 245 (19.58%) | 1.41 [1.09 - 1.81] |
|  | 5th | 763 (80.15%) | 189 (19.85%) | 1.30 [1.00 - 1.69] |
| Indigenous | ***≤1/2 minimum wage vs >1/2 minimum wage*** | | | |
|  | 1st | 872 (87.99%) | 119 (12.01%) | 1.00 (Base) |
|  | 2nd | 1,033 (87.84%) | 143 (12.16%) | 1.03 [0.73 - 1.46] |
|  | 3rd | 1,354 (83.48%) | 268 (16.52%) | 1.46 [0.96 – 2.23] |
|  | 4th | 1,321 (84.41%) | 244 (15.59%) | 1.57 [0.95 - 2.61] |
|  | 5th | 1,670 (85.64%) | 280 (14.36%) | 1.37 [1.00 - 1.87] |

* Adjusted for age, sex, education, experiencing homelessness, HIV, alcohol abuse, illicit drug use, tobacco use, clinical form of TB, diabetes, mental health/cognitive developmental conditions, DOTS.

** Test for linearity using likelihood ratio test was <0.0001 for both racial and income segregation

OR: Odds Ratio

95%CI: 95% Confidence Interval

**Supplementary Table 3. Baseline distribution of income and racial segregation indices (n= 656,816)**

| Residential segregation indices | N | Mean | SD | Median | IQR |
| --- | --- | --- | --- | --- | --- |
| *≤1/2 minimum wage vs >1/2 minimum wage* | | | | | |
| 1st quintile | 130,568 | 0.22 | 0.32 | 0.22 | 0.04 |
| 2nd quintile | 131,401 | 0.28 | 0.02 | 0.28 | 0.03 |
| 3^rd^ quintile | 133,373 | 0.33 | 0.01 | 0.32 | 0.18 |
| 4th quintile | 140,463 | 0.37 | 0.01 | 0.37 | 0.03 |
| 5th quintile | 121,011 | 0.42 | 0.02 | 0.41 | 0.01 |
| *Black & Mixed/Brown vs. White* | | | | | |
| 1st quintile | 131,379 | 0.22 | 0.03 | 0.22 | 0.04 |
| 2nd quintile | 131,242 | 0.28 | 0.01 | 0.28 | 0.01 |
| 3^rd^ quintile | 131,765 | 0.31 | 0.01 | 0.32 | 0.03 |
| 4th quintile | 133,198 | 0.35 | 0.01 | 0.36 | 0.01 |
| 5th quintile | 129,232 | 0.40 | 0.03 | 0.39 | 0.03 |

SD: Standard deviation

IQR: Interquartile range

**Supplementary Table 4. Crude and multivariable logistic regression between household income and racial segregation and loss to follow up of TB treatment**

| Residential segregation indices | | OR (95% CI)^1^  N= 602,450 | Adjusted OR (95% CI)^2^  N=602,450 |
| --- | --- | --- | --- |
| *Income segregation (N, %unfavourable TB treatment outcomes)* | | | |
| ≤1/2 minimum wage vs. >1/2 minimum wage | 1st quintile  (1,909, 11.84%) | 1.00 (Base) | 1.00 (Base) |
|  | 2nd quintile  (2,249, 13.94%) | 0.97 (0.95 – 1.00) | 0.96 (0.94 – 0.99) |
|  | 3rd quintile  (2,589, 16.05%) | 1.18 (1.15 – 1.21) | 1.20 (1.17 – 1.23) |
|  | 4th quintile  (7,916, 49.08%) | 1.39 (1.36 – 1.42) | 1.45 (1.42 – 1.49) |
|  | 5th quintile  (1,467, 9.09%) | 1.65 (1.61 – 1.70) | 1.57 (1.53 – 1.61) |
| *Racial segregation* | | | |
| Black & Mixed/Brown vs. White | 1st quintile  (1,950, 12.09%) | 1.00 (Base) | 1.00 (Base) |
|  | 2nd quintile  (3,095, 19.19%) | 0.96 (0.94 – 0.99) | 0.99 (0.96 – 1.01) |
|  | 3rd quintile  (2,136, 13.24%) | 0.97 (0.95 – 1.00) | 0.94 (0.92 – 0.96) |
|  | 4th quintile  (8,058, 49.96%) | 1.21 (1.18 – 1.24) | 1.25 (1.22 – 1.28) |
|  | 5th quintile  (891, 5.52%) | 1.34 (1.31 – 1.37) | 1.22 (1.19 – 1.25) |

^1^Including a priori (age, sex)

^2^Adjusted for age, sex, education, experiencing homelessness, HIV, alcohol abuse, illicit drug use, tobacco use, diabetes, mental health/cognitive developmental conditions, DOTS

OR: Odds Ratio

95%CI: 95% Confidence Interval

**Supplementary Table 5. Complete-case analysis of crude and multivariable logistic regression between household income and racial segregation and TB treatment outcome**

| Residential segregation indices | | OR (95% CI)^1^  N= 111,838 | Adjusted OR (95% CI)^2^  N=111,838 |
| --- | --- | --- | --- |
| *Income segregation (N, %unfavourable TB treatment outcomes)* | | | |
| ≤1/2 minimum wage vs. >1/2 minimum wage | 1st quintile  (1,909, 11.84%) | 1.00 (Base) | 1.00 (Base) |
|  | 2nd quintile  (2,249, 13.94%) | 1.34 (1.25 - 1.43) | 1.27 (1.19 - 1.36) |
|  | 3rd quintile  (2,589, 16.05%) | 1.37 (1.29 - 1.46) | 1.31 (1.23 - 1.40) |
|  | 4th quintile  (7,916, 49.08%) | 1.91 (1.81 - 2.02) | 1.52 (1.44 - 1.61) |
|  | 5th quintile  (1,467, 9.09%) | 1.74 (1.62 – 1.87) | 1.52 (1.41 - 1.64) |
| *Racial segregation* | | | |
| Black & Mixed/Brown vs. White | 1st quintile  (1,950, 12.09%) | 1.00 (Base) | 1.00 (Base) |
|  | 2nd quintile  (3,095, 19.19%) | 1.43 (1.35 - 1.52) | 1.42 (1.33 - 1.51) |
|  | 3rd quintile  (2,136, 13.24%) | 1.42 (1.33 - 1.52) | 1.33 (1.25 - 1.43) |
|  | 4th quintile  (8,058, 49.96%) | 1.87 (1.77 - 1.97) | 1.50 (1.42 - 1.59) |
|  | 5th quintile  (891, 5.52%) | 1.89 (1.74 – 2.06) | 1.65 (1.51 - 1.81) |

^1^Including a priori (age, sex)

^2^Adjusted for age, sex, education, experiencing homelessness, HIV, alcohol abuse, illicit drug use, tobacco use, clinical form of TB, diabetes, mental health/cognitive developmental conditions, DOTS excluding missing data.

OR: Odds Ratio

95%CI: 95% Confidence Interval
